# Supplementary material for: Microplastic-Free Microcapsules to Encapsulate Health-Promoting Limonene Oil
Source: Molecules. 2022 Oct 25;27(21):7215. doi: 10.3390/molecules27217215 (PMC9659182; doi:10.3390/molecules27217215)
Supplement: Supplementary file 1 [file molecules-27-07215-s001.zip › molecules-1982586_Supplementary Material.pdf]

**Supplementary Material**

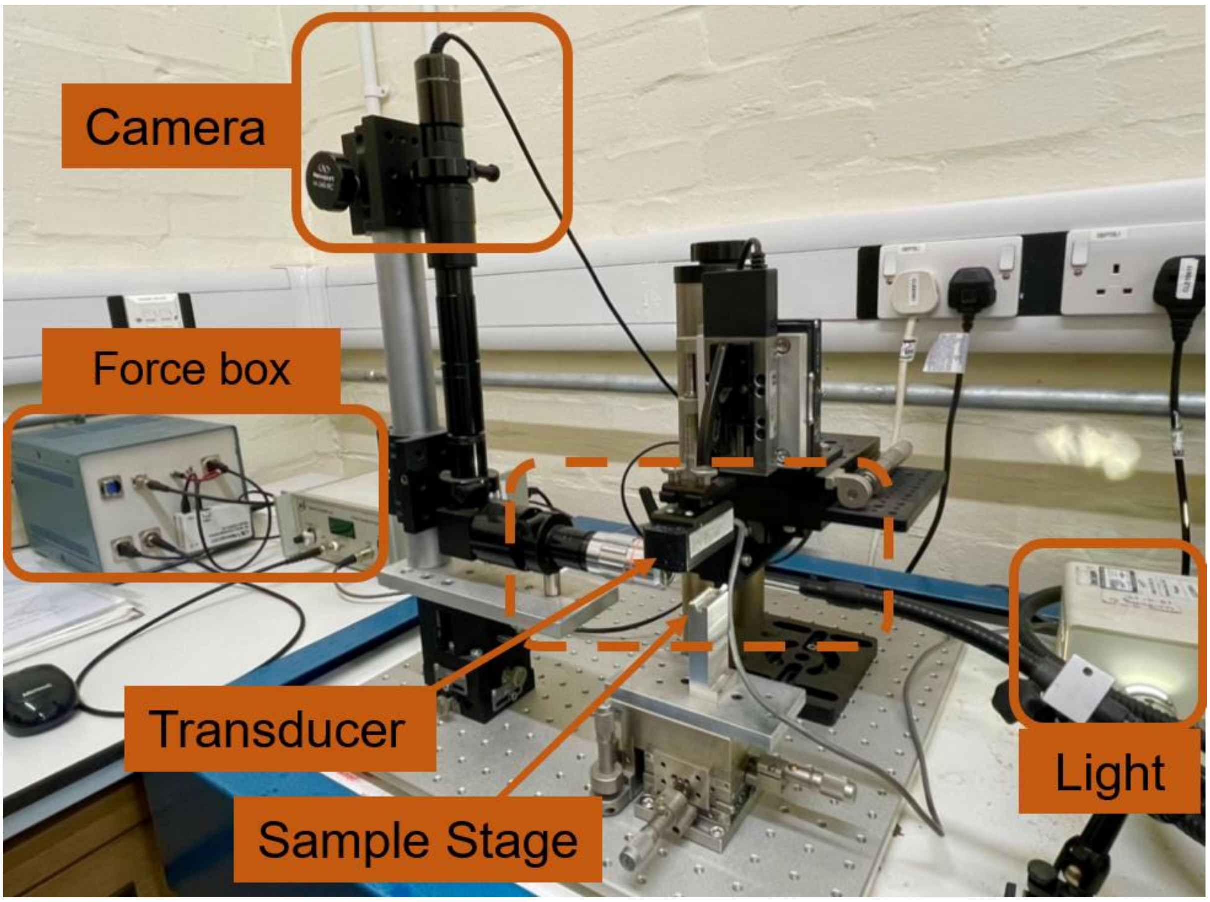


**Figure S1.** Semiautomatic micromanipulation rig (University of Birmingham, England, UK).

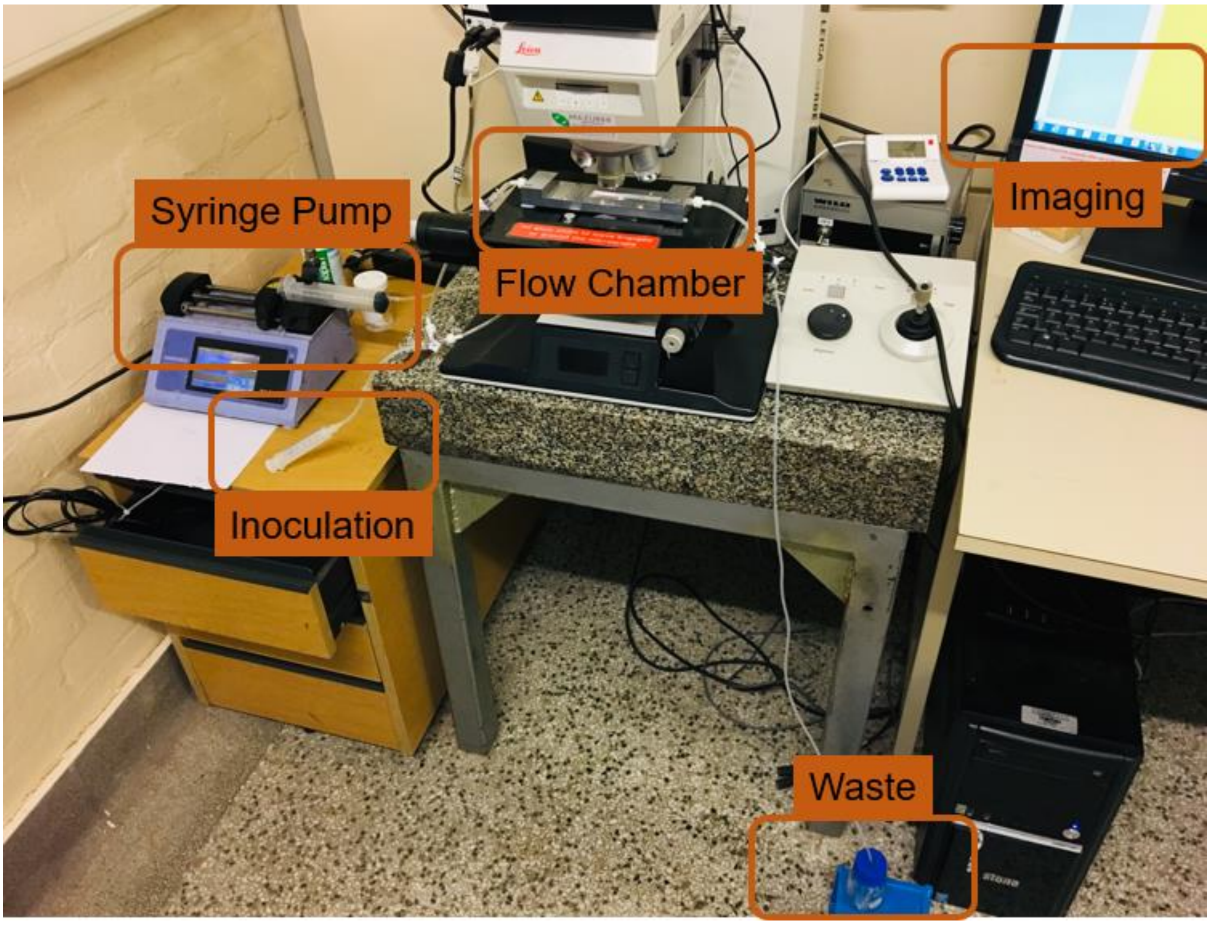


**Figure S2.** Microfluidic flow chamber apparatus (University of Birmingham, England, UK).
